# Six Months After the Patients Stayed Home: A Nationwide Study of Cardiac Mortality and Readmissions Following the First Wave of the COVID-19 Pandemic in Malta

**DOI:** 10.7759/cureus.23065

**Published:** 2022-03-11

**Authors:** Neil Grech, Rachel A Xuereb, Robert G Xuereb, Maryanne Caruana

**Affiliations:** 1 Department of Cardiology, Mater Dei Hospital, Msida, MLT; 2 Department of Medicine, University of Malta, Msida, MLT

**Keywords:** patient readmission, mortality, acute coronary syndrome, sars-cov-2, covid-19

## Abstract

Background

The first COVID-19 wave resulted in a significant decline in acute cardiac admissions (ACAs) and delays to hospital presentation in Malta, as well as an excess of out-of-hospital cardiac arrests. The aim was to investigate the impact of the observed delays in presentation in 2020 on mortality and cardiac readmissions at six months.

Methods

All ACAs between 28th February and 30th April 2020 (first wave of COVID-19 in Malta) were included, and the corresponding 2019 period was used as a control. ACA was defined as an unplanned admission of an adult (aged ≥16 years) under the care of a cardiologist. Outcomes over the six months following the index ACA included death, cardiac readmission, and planned cardiac intervention at discharge. The term ‘death’ referred to all-cause mortality. Cardiac readmissions referred to unplanned admissions for acute cardiac pathology following the index ACA. During sub-analyses, ACAs were divided into acute coronary syndrome (ACS) and non-ACS. A first analysis compared the frequency of deaths, cardiac readmissions, and planned interventions between the 2019 and 2020 cohorts. A second analysis investigated differences in six-month survival and freedom from readmission between the two cohorts. Both analyses were followed by a sub-analysis.

Results

There were 330 ACAs among the 2019 cohort and 220 in 2020. There were no significant differences between the 2019 and 2020 cohorts in all-cause mortality (2019, 8.8% vs 2020, 8.2%, p=0.466) and Kaplan-Meier survival estimates at a six-month follow-up (2019, 169.06 days (95% CI 164.95-173.17) vs 2020, 168.27 days (95% CI 162.82-173.72), p=0.836), including subgroup analysis for non-ACS (2019, 168.52 days (95% CI 163.08-173.96) vs 168.11 days (95% CI 160.93-175.30), p=0.952) and ACS patients (169.81 days (95% CI 163.54-176.09) in 2019 vs 168.45 days (95% CI 160.17-176.73) in 2020, p=0.739).

A significantly higher number of patients from the 2019 cohort (75/319, 23.5%) required readmission compared to 2020 (32/212; 15.1%) (p=0.02). Similarly, there was shorter freedom from cardiac readmission among 2019 patients (mean 150.98 days (95% CI 144.63-157.33)) compared to 2020 patients (mean 158.66 days (95% CI 151.58-165.74, p=0.024). During sub-analysis, the difference in freedom from readmission was significant only for non-ACS patients (mean of 145.45 days (95% CI 136.58-154.32) in 2019 vs 158.92 days (95% CI 149.19-168.64) in 2020, p=0.018).

Analysis of cardiac interventions during the six months post-index ACA discharge showed significantly more planned cardiac interventions in 2019 (52/319; 16.3%) compared to 2020 (20/212; 9.4%) (p=0.027).

Conclusions

A delay in presentation of ACAs during COVID-19 in Malta resulted in lower readmission rates and increased freedom from readmissions, with no excess in all-cause mortality at a six-month follow-up. The reasons for the optimistic outcomes of patients admitted during the first wave of COVID-19 may be multifactorial. Reasons may include ongoing fear of hospital presentation, a more holistic approach to patients’ in-hospital care during 2020 aimed at reducing further hospital contact post-discharge, and a selection bias secondary to an excess of out-of-hospital cardiac arrests during the initial wave of COVID-19. Further studies will be required to truly assess the collateral impact of non-COVID-19-related illness. Public education on cardiovascular health is vital and must be emphasized during the pandemic.

## Introduction

Illness caused by the severe acute respiratory syndrome coronavirus 2 (SARS-CoV-2), COVID-19, has become a global emergency, with millions of cases confirmed worldwide. Despite widespread healthcare system efforts to continue providing inpatient and outpatient care while dealing with the increased load of COVID-19 admissions, many institutions observed declines and delays in acute medical and surgical presentations [1-3], most likely reflecting a fear of contracting the SARS-CoV-2 virus from hospital environments, particularly during the peaks of the pandemic waves [2-5]. The longer-term implications of this phenomenon remain to be ascertained.

The first case of SARS-CoV-2 infection in Malta was reported in early March 2020 and declines in hospital admissions started being observed in subsequent weeks. The first study by our group investigated the initial impact of the first wave of the COVID-19 pandemic on acute cardiac admissions (ACAs) and early cardiac mortality in Malta and documented a significant decline in ACAs and delays in presentation as well as an excess of out-of-hospital cardiac arrests when compared to the same nine-week period of 2019 [6]. Health services in Malta are free at the point of care and funded through taxation and national insurance, therefore, the financial burden of the pandemic should not have impeded patients’ access to cardiovascular care. The aims of the current study were to investigate the impact of the observed delays in presentation during 2020 on mortality and cardiac readmissions at a six-month follow-up.

## Materials and methods

This population-based study consisted of all patients admitted with acute cardiac pathology to Mater Dei Hospital (MDH) during the nine-week period between 28th February and 30th April 2020, while the control cohort was made up of all ACAs during the corresponding calendar period of the previous year (27th February - 30th April 2019). Malta is a small country in Southern Europe with a population of approximately half a million. MDH is the only center in Malta that provides a 24/7 percutaneous coronary intervention (PCI) service, along with other specialized cardiac services which remained operational throughout COVID-19. The hospital’s catchment area includes all of Malta and the smaller sister island of Gozo.

Three outcomes: (a) death, (b) cardiac readmission, and (c) planned cardiac intervention at discharge following index admission, were recorded during the six-month period following the index ACA. Data for all outcomes was obtained from MDH’s electronic patient management and discharge summary databases and was recorded prospectively for patients in the 2020 cohort and retrospectively for those in the 2019 control cohort. Following institutional data protection clearance, the study was approved by the University of Malta Research Ethics Committee, in compliance with the ethical guidelines of the 1975 Declaration of Helsinki and was performed with a waiver of consent.

As per our original paper [6], ACA was defined as an unplanned admission of an adult subject (aged ≥16 years) under the care of the MDH cardiology team for the management of acute cardiac pathology and included all admissions through MDH Accident and Emergency Department, direct admissions from cardiology outpatient clinics and emergency inpatient transfers from the regional hospital in Gozo. The term ‘death’ referred to all-cause mortality. Cardiac readmissions referred to unplanned admissions for acute cardiac pathology following the index ACA. For patients with multiple cardiac readmissions, only the first readmission after the index ACA was considered for analysis purposes. Planned cardiac interventions referred to any percutaneous or surgical cardiac procedure that was part of the subject’s discharge plan following the index ACA. These included coronary interventions of bystander or non-culprit coronary lesions, pacemaker and defibrillator therapy device implantations, electrophysiological procedures, valvular interventions and revascularization and valvular surgery. Patients that died during their index ACA were automatically excluded from any analyses on subsequent cardiac readmissions or interventions. During sub-analyses, ACAs were divided into two broad categories: acute coronary syndrome (ACS) and non-ACS. ACS referred to all forms of acute coronary events and included ST segment elevation myocardial infarction (STEMI) and non-ST segment elevation ACS (NSTE-ACS), with or without biomarker rise (non-STEMI and unstable angina, respectively). Non-ACS ACAs referred to all other admissions not directly related to an acute coronary event and included heart failure, arrhythmias, and valvular disease.

A first analysis compared the frequency of deaths, cardiac readmissions, and planned interventions between the 2019 and 2020 cohorts. A second analysis investigated differences in six-month survival and freedom from readmission between the two cohorts, followed by a sub-analysis by admission category (ACS/non-ACS). Categorical variables were analyzed using the Chi-square test, while Fisher’s exact test was applied in the case of smaller sample sizes. Comparison of all continuous variables was performed using the Mann-Whitney U test after the Shapiro-Wilk test established a non-normal distribution. Estimates of survival and freedom from readmission were assessed with Kaplan-Meier curves using the log-rank test to compare estimates. All analyses were performed using SPSS 26 (IBM SPSS 26, IBM Corp., Armonk, USA). Statistical significance was defined as p<0.05.

## Results

There were 330 patients in the 2019 cohort and 220 in the 2020 cohort. There were no significant differences in gender (males 2019: 237, 71.8% vs. 2020: 163, 74.1%; p=0.625) or age at index ACA (median age 2019: 68 years (IQR 17) vs. 2020: 67 years (IQR 17); p=0.115). Equally, there was no significant difference in the admission category, with ACS being the indication for ACA in 41.8% of patients in 2019 and 47.7% of patients in the 2020 cohort (p=0.19).

**Figure 1:**
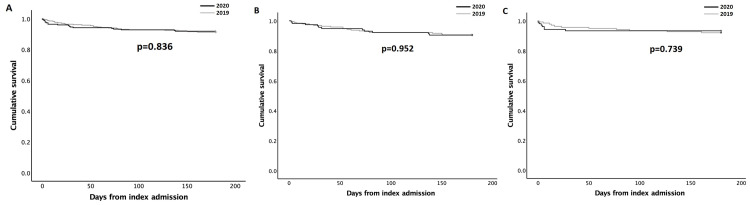
Kaplan-Meier six-month survival estimates comparing 2019 and 2020 cohorts (A), and subgroup analysis of patients admitted with non-ACS (B) and ACS (C) pathologies ACS: Acute coronary syndrome

Contrary to expectations, despite the previously reported significant delays in presentation in 2020 [6], there was no significant excess of all-cause deaths at six-month follow-up between the two cohorts, with 29 (8.8%) deaths in 2019 compared to 18 (8.2%) deaths in 2020 (p=0.466). Similarly, Kaplan-Meier six-month survival estimates were comparable between the two cohorts, with a mean survival estimate of 169.06 days (95% CI 164.95-173.17) from index admission in 2019 compared to 168.27 days (95% CI 162.82-173.72) in 2020 (p=0.836) (Figure 1A). Equally, no survival differences were observed during admission subgroup analyses, with a mean survival estimate of 168.52 days (95% CI 163.08-173.96) in 2019 compared to 168.11 days (95% CI 160.93-175.30) in 2020 (p=0.952) for patients admitted with non-ACS (Figure 1B) and a mean survival estimate of 169.81 days (95% CI 163.54- 176.09) in 2019 compared to 168.45 days (95% CI 160.17-176.73) in 2020 (p=0.739) for ACS pathology (Figure 1C).

**Figure 2:**
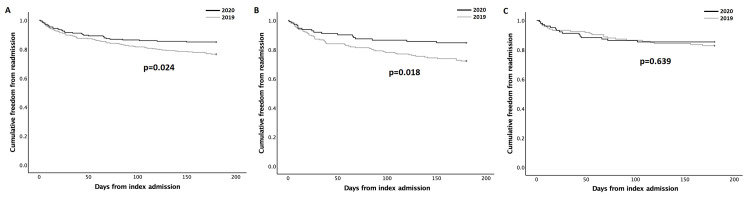
Kaplan-Meier six-month freedom from cardiac readmission estimates of 2019 and 2020 patients (A), and sub-analysis of patients admitted with non-ACS (B) and ACS (C) pathologies. ACS: Acute coronary syndrome

A significantly higher number of patients from the 2019 cohort (75/319; 23.5%) experienced cardiac readmission during the six-month follow-up period compared to 32/212 (15.1%) patients in the 2020 cohort (p=0.02). Similarly, there was a shorter freedom from cardiac readmission among 2019 (mean estimate 150.98 days (95% CI 144.63-157.33)) (p=0.024) compared to 2020 patients (mean estimate 158.66 days (95% CI 151.58-165.74) (Figure 2A). During sub-analysis by admission category, the difference in freedom from cardiac readmission was only significant for patients with a non-ACS index ACA, with a mean estimate of 145.45 days (95% CI 136.58-154.32) in 2019 and a mean estimate of 158.92 days (95% CI 149.19-168.64) in 2020 (p=0.018) (Figure 2B). Freedom from cardiac readmission for patients with an ACS index ACA was comparable in both cohorts (mean estimate 158.71 days (95% CI 150.05-167.36) in 2019 vs 158.28 days (95% CI 148.07-168.70) in 2020 (p=0.639) (Figure 2C).

Significantly more patients among the 2019 cohort (52/319; 16.3%) left hospital with a planned cardiac intervention compared to 2020 (20/212; 9.4%) (p=0.027).

## Discussion

A delay in presentation of ACAs during COVID-19 in Malta has resulted in lower readmission rates and increased freedom from readmissions, with no excess in all-cause mortality at a six-month follow-up. The importance of timely presentation and management of acute cardiac pathology is well-known and is particularly recognized in acute STEMI, with delays in revascularization translating into increased morbidity and mortality [7,8]. It is reasonable to postulate that the excess mortality related to the COVID-19 pandemic that has been observed in many countries could be partly explained by delays in hospital presentation among patients with acute pathology, including acute cardiac conditions [9-16]. In line with these observations, our initial study demonstrated an excess of out-of-hospital cardiac deaths during the early months of the first wave of the COVID-19 pandemic [6]. However, contrary to expectation, the significant delays to presentation among our institution’s 2020 acute cardiac admission patients failed to translate into an excess of mortality at six-month follow-up, which differs from what was documented by other researchers [17-19]. Primessnig et al. (2020) evaluated the impact of COVID-19 on acute myocardial infarction admissions presenting during the first four months of 2020 compared to the previous year. A significant decline and delay in time from symptom onset to first medical contact of cardiac admissions translated into higher inpatient mortality, with a poorer cardiac outcome upon follow-up, with higher N-terminal pro-brain natriuretic peptide (NT-proBNP) levels and a lower ejection fraction in patients admitted during early COVID-19 period [17]. The worse outcome in myocardial infarction patients reported above was echoed in other studies where the delay in presentation of ACS patients resulted in higher in-hospital deaths, life-threatening arrhythmias, cardiogenic shock and mechanical circulatory support when compared to pre-COVID admissions [18,19].

One possible explanation is an inadvertent selection bias resulting from the 2020 highest risk acute cardiac patients, with an inherent likelihood of rapid deterioration and poorer outcome, never actually presenting to our institution in the first place but instead succumbing to out-of-hospital death due to their delayed presentation. This explanation is supported by our earlier finding of a significant excess of out-of-hospital cardiac deaths in 2020 when compared to the same calendar weeks of 2019 [6]. Secondly, it could be argued that six months might be too short a follow-up period to demonstrate excess mortality resulting from delayed presentation of acute cardiac pathology and that a clearer picture might be obtained at later follow-up.

The other unexpected finding in our study was that of fewer readmissions and longer freedom from readmission among patients in the 2020 cohort. This could be partly explained by the fact that the local decline in overall admissions and the reduction in elective procedures during the early stages of the COVID-19 pandemic gave clinicians time to manage patients more comprehensively and to discharge them without any significant outstanding issues, making it less likely for them to represent. This is supported by the documented lower planned intervention rate at discharge among our study cohort patients. Also, during the first months of the COVID-19 pandemic, the majority of outpatient cardiology follow-ups in our institution were being performed by means of phone consultations and this could have resulted in some patients’ symptoms not being fully appreciated, hence reducing the number of readmissions triggered from the clinic. Ultimately, it is the authors’ opinion that a significant contributor to the lower readmissions in 2020 was the ongoing hesitancy among patients to come to the hospital due to fear of contracting SARS-CoV-2, especially as community cases increased [1-3,6].

The major limitation of this study was the low patient numbers limiting the power of our results. To offset this, we limited the analysis of patient subgroups to avoid reducing the numbers further. Further to this, we relied on online databases for retrospective data analysis, occasionally resulting in the absence of some data.

## Conclusions

Despite significant delays in presentation of ACAs during the early stages of COVID-19 in Malta, our institution demonstrated no excess all-cause mortality and a reduction in cardiac readmissions at six-month follow-up when compared to the same period in 2019. This finding may be confounded by patients’ ongoing hesitancy to present to the hospital, as well as a more holistic in-patient approach during 2020 aimed at reducing further hospital contact post-discharge. Although the findings of the study portray a positive outcome for patients admitted during the first wave of COVID-19, one must consider the patients who failed to present during the initial wave and subsequently suffered out-of-hospital cardiac arrests. The true scale of the excess non-COVID-19-illness-related morbidity and mortality will require studies on a longer-term basis. Institutions must aim to reassure the public that hospital environments are safe and that cardiovascular care remains available and essential.
